# Intercellular mitochondrial transfer in melanoma progression and therapeutic resistance: mechanisms and targeting potential

**DOI:** 10.3389/fonc.2026.1835726

**Published:** 2026-05-21

**Authors:** Qingqing Yan, Tao Sun, Kang Chen, Hao Hu

**Affiliations:** The First People’s Hospital of Wenling, Wenling, China

**Keywords:** immune regulation, intercellular mitochondrial transfer, melanoma, oxidative phosphorylation, therapeutic resistance, tumor microenvironment

## Abstract

Intercellular mitochondrial transfer has emerged as a novel mode of metabolic communication, enabling the exchange of functional mitochondria and their associated components between cells via tunneling nanotubes, extracellular vesicles, and direct cell–cell contact. Melanoma is a highly aggressive malignancy characterized by remarkable metabolic plasticity, in which disease progression and therapeutic resistance are closely linked to mitochondrial reprogramming. Accumulating evidence indicates that, under conditions of therapeutic pressure or metabolic impairment, melanoma cells can acquire exogenous mitochondria to restore oxidative phosphorylation (OXPHOS), maintain redox homeostasis, and enhance survival. This process contributes to resistance to targeted therapies, immune evasion, and increased invasive and metastatic potential. Conversely, in specific contexts, intercellular mitochondrial transfer may exert tumor-suppressive effects by enhancing the metabolic fitness of immune cells, activating innate immune signaling pathways, or inducing oxidative stress–mediated apoptosis. These findings underscore the context-dependent nature of its biological effects, which are governed by factors such as donor and recipient cell identity, mitochondrial integration status, and microenvironmental stress conditions. In this review, we systematically summarize the principal mechanisms of intercellular mitochondrial transfer and highlight its bidirectional roles in melanoma progression and therapeutic resistance. Furthermore, we propose a context-dependent regulatory framework and discuss potential intervention strategies. A deeper understanding of this process may provide new theoretical insights for integrating metabolic modulation with targeted and immunotherapeutic approaches in precision melanoma treatment.

## 1 Introduction

Melanoma is a highly aggressive malignant tumor originating from melanocytes (1). Although it accounts for a relatively small proportion of skin cancer cases, it is responsible for the majority of skin cancer–related deaths (2, 3). In recent years, the global incidence of melanoma has continued to rise, with particularly notable increases in regions with high ultraviolet exposure and among younger populations (4, 5). Early-stage melanoma can often be effectively treated through surgical excision, achieving a high cure rate; however, once regional or distant metastasis occurs, patient prognosis deteriorates significantly (6–8). Advanced melanoma is characterized by pronounced heterogeneity, strong metastatic potential, and remarkable adaptability to therapeutic pressure, making it one of the most challenging solid tumors in contemporary oncology (9, 10). Therefore, elucidating the molecular basis underlying melanoma progression and therapeutic failure is critical for improving long-term patient survival.

In recent years, targeted therapy and immunotherapy have substantially reshaped the therapeutic landscape of advanced melanoma (11). However, both primary and acquired resistance are almost inevitable, whether in the context of BRAF/MEK inhibitor combinations or immune checkpoint blockade (12, 13). Key mechanisms driving resistance include pathway reactivation, epigenetic reprogramming, immune evasion, and remodeling of the tumor microenvironment (14–16). Increasing evidence further highlights metabolic plasticity as a central driver enabling melanoma cells to adapt to therapeutic stress (10). In particular, mitochondrial reprogramming and enhanced oxidative phosphorylation (OXPHOS) have been strongly associated with resistance to targeted therapies and failure of immunotherapy (17, 18).

Mitochondria, as central organelles in cellular energy metabolism, are primarily responsible for ATP production and redox regulation (19). Emerging studies have revealed that mitochondria can be transferred between cells via tunneling nanotubes, extracellular vesicles, or direct cell–cell contact, a phenomenon referred to as intercellular mitochondrial transfer (20–22). Transferred mitochondria not only provide metabolic capacity but also carry mitochondrial DNA and redox-related signaling molecules, thereby reshaping the metabolic state and immune responses of recipient cells (23, 24). In various solid tumors, this process has been shown to enhance tumor cell survival and promote therapeutic resistance (25). Notably, under certain contexts, mitochondrial transfer may exert effects opposite to tumor promotion, reflecting pronounced context dependency and functional bidirectionality (26).

Although research on intercellular mitochondrial transfer in tumor biology has been expanding, a systematic understanding of its role in melanoma—an archetypal tumor highly dependent on metabolic adaptation and immune regulation—remains lacking. Therefore, this review aims to comprehensively summarize the molecular mechanisms of intercellular mitochondrial transfer and its roles in melanoma progression and therapeutic resistance. We further propose a context-dependent regulatory framework and discuss its potential as a novel therapeutic and investigative target, with the goal of providing new theoretical insights for precision treatment strategies in melanoma.

## 2 Current therapeutic landscape and resistance in melanoma

The current management of melanoma has evolved into a comprehensive, multimodal strategy centered on surgery, targeted therapy, and immunotherapy. However, durable disease control remains limited in patients with advanced-stage disease. Although existing treatment modalities can achieve clinical benefits at specific stages, their limitations become particularly pronounced in metastatic melanoma (see **Table 1**) (15, 27–39).

**Table 1:** Overview of current therapeutic strategies for melanoma.

Treatment modality	Advantages	Limitations	References
Surgical resection	Suitable for early-stage patients; enables complete tumor removal with curative potential	Limited to localized disease; minimal benefit for advanced or metastatic patients	(27–29)
Chemotherapy	Applicable to advanced-stage patients; may provide symptomatic relief in some cases	Low response rates; significant systemic toxicity; high propensity for resistance	(15, 30, 31)
Radiotherapy	Effective for brain metastases or local lesion control	Limited efficacy in controlling systemic disease	(32, 33)
Targeted therapy	High initial response rates in mutation-positive patients	Acquired resistance is common; active metabolic compensation mechanisms	(34–36)
Immunotherapy	Can confer durable survival benefits	Primary and acquired resistance; pronounced immune evasion	(37–39)

In the absence of substantial therapeutic intervention, melanoma cells typically maintain concurrent activity of glycolysis and OXPHOS (40). This metabolic profile is closely linked to their differentiation state: subpopulations with high expression of microphthalmia-associated transcription factor (MITF) generally exhibit enhanced mitochondrial respiration, whereas dedifferentiated cells preferentially rely on glycolytic metabolism (41, 42). Under therapeutic or stress conditions, this metabolic state can undergo predictable reprogramming. Experimental studies in mouse models have shown that treatment with BRAF/MEK inhibitors induces upregulation of the MITF–PGC1α axis in a subset of melanoma cells, promoting mitochondrial biogenesis and the expression of respiratory chain complexes, thereby increasing OXPHOS dependency (43). This process is accompanied by elevated reactive oxygen species (ROS) production and alterations in the NAD^+^/NADH ratio, which in turn modulate key cell survival–related signaling pathways, particularly the PI3K–AKT, MAPK/ERK, and NF-κB cascades (44–46). Further evidence indicates that cells with higher OXPHOS activity are more likely to survive sustained therapeutic pressure and progressively emerge as a major source of acquired resistance (47). In addition, enhanced mitochondrial respiration can influence the efficacy of immunotherapy by altering local oxygen utilization and redox balance (48).

Melanoma exhibits a high degree of metabolic plasticity under therapeutic pressure. Resistance to both targeted therapy and immunotherapy is closely associated with enhanced mitochondrial function and increased reliance on OXPHOS (49, 50). These observations suggest that mitochondrial functional remodeling constitutes a fundamental mechanism by which tumor cells adapt to therapeutic stress. However, current studies on treatment resistance in melanoma remain largely focused on the reactivation of signaling pathways and the regulation of immune checkpoints. In contrast, metabolic reprogramming—particularly its dynamic regulation within the tumor microenvironment—remains insufficiently characterized. Beyond serving as central regulators of intracellular metabolism, mitochondria may also participate in metabolic and immune modulation within the tumor microenvironment through intercellular information transfer (48). This phenomenon of intercellular mitochondrial transfer may provide a novel perspective for understanding the adaptive evolution of melanoma and the development of therapeutic resistance.

## 3 Intercellular mitochondrial transfer

**Figure 1:**
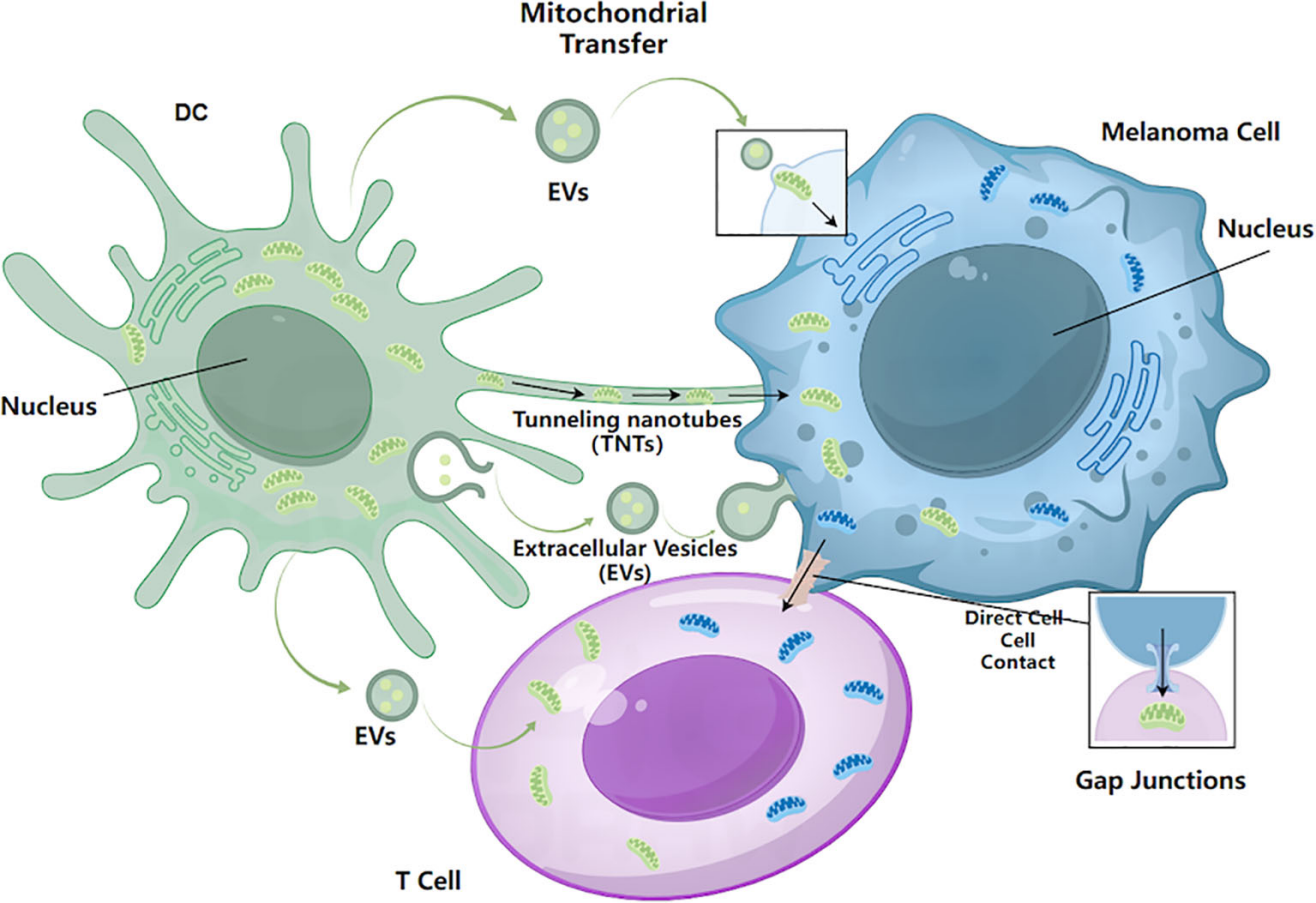
Major mechanisms of intercellular mitochondrial transfer. Mitochondria can be exchanged between tumor cells and surrounding stromal or immune cells via three principal routes: (i) TNTs, which enable direct cytoplasmic continuity and organelle trafficking between connected cells; (ii) EVs, which mediate the transfer of mitochondrial components or intact mitochondria through vesicle uptake; and (iii) direct cell–cell contact, including gap junction–associated transfer. These mechanisms collectively facilitate dynamic and context-dependent mitochondrial exchange among melanoma cells and immune cells.

Intercellular mitochondrial transfer refers to the movement of intact or partial mitochondrial structures between cells through specialized conduits, where they exert functional regulatory effects within recipient cells (51). This process involves not only the restoration of metabolic function but also the transfer of mitochondrial DNA (mtDNA), metabolic enzyme complexes, and redox signaling molecules (52–54). From a systems perspective, intercellular mitochondrial transfer can be viewed as a unique form of metabolic information flow, with potential roles in maintaining tissue homeostasis, facilitating injury repair, and enabling adaptation to stress (55, 56). This highlights that mitochondrial function possesses regulatory properties beyond the single-cell level, offering a novel conceptual framework for understanding metabolic regulation in complex diseases. The principal routes and basic modes of transfer are illustrated in **Figure 1**.

Current evidence indicates that intercellular mitochondrial transfer can occur via multiple mechanisms involving either direct physical connections or vesicle-mediated pathways. Broadly, these mechanisms can be categorized into three main types: direct cell–cell connections, vesicle-mediated transfer, and contact-dependent exchange (57). Among direct structural connections, tunneling nanotubes (TNTs) represent the most well-characterized conduits (58). Composed primarily of cytoskeletal elements such as F-actin, TNTs form continuous bridges between adjacent or even spatially separated cells (25). Through these structures, organelles—including mitochondria—can be transported bidirectionally. TNT-mediated mitochondrial transfer is most prominent under conditions of stress, hypoxia, or cellular injury, and is particularly active within dynamic pathological environments such as the tumor microenvironment (20, 59). Thus, TNTs primarily facilitate efficient, short-range, direct organelle transfer.

Extracellular vesicles (EVs) constitute another major vehicle for intercellular mitochondrial communication. EVs—including exosomes and microvesicles—can encapsulate mitochondrial fragments, mitochondrial proteins, and even mtDNA, and release them into the extracellular space (60, 61). Recipient cells internalize these vesicles via endocytosis or membrane fusion, thereby acquiring mitochondrial components that can alter cellular function. In contrast to the short-range transport mediated by TNTs, EV-based transfer is better suited for long-distance and indirect dissemination of mitochondria-related signals.

In addition, under certain conditions, transient membrane contacts or partial cytoplasmic fusion between cells may enable the direct exchange of mitochondria. Although the precise molecular mechanisms governing this process remain incompletely understood, it is considered an important complementary route of intercellular mitochondrial transfer. Mitochondrial positioning and trafficking depend on the microtubule network and motor proteins, while specific outer mitochondrial membrane proteins and transport regulators play critical roles in mitochondrial loading, trafficking, and release (62–64). Under stress conditions, cells may selectively enhance or restrict mitochondrial transfer by modulating these molecular mechanisms, thereby contributing to the dynamic remodeling of local metabolic and signaling networks.

Although intercellular mitochondrial transfer has attracted increasing attention in cancer research, it was originally identified in the context of tissue repair and regenerative medicine. Under physiological conditions, this process likely serves multiple beneficial functions. During tissue injury or hypoxia, healthy cells can donate functional mitochondria to damaged cells, restoring respiratory chain activity and ATP production, thereby promoting tissue repair (65, 66). Within stem cell niches, mitochondrial transfer may participate in regulating stem cell differentiation and metabolic homeostasis (67). In the immune system, mitochondrial status directly influences immune cell effector functions and inflammatory responses, and intercellular transfer may contribute to the maintenance of immune homeostasis (68, 69). However, within the melanoma microenvironment, this inherently protective physiological mechanism may become dysregulated, thereby contributing to disease progression.

## 4 Bidirectional biological roles of intercellular mitochondrial transfer in melanoma

**Figure 2:**
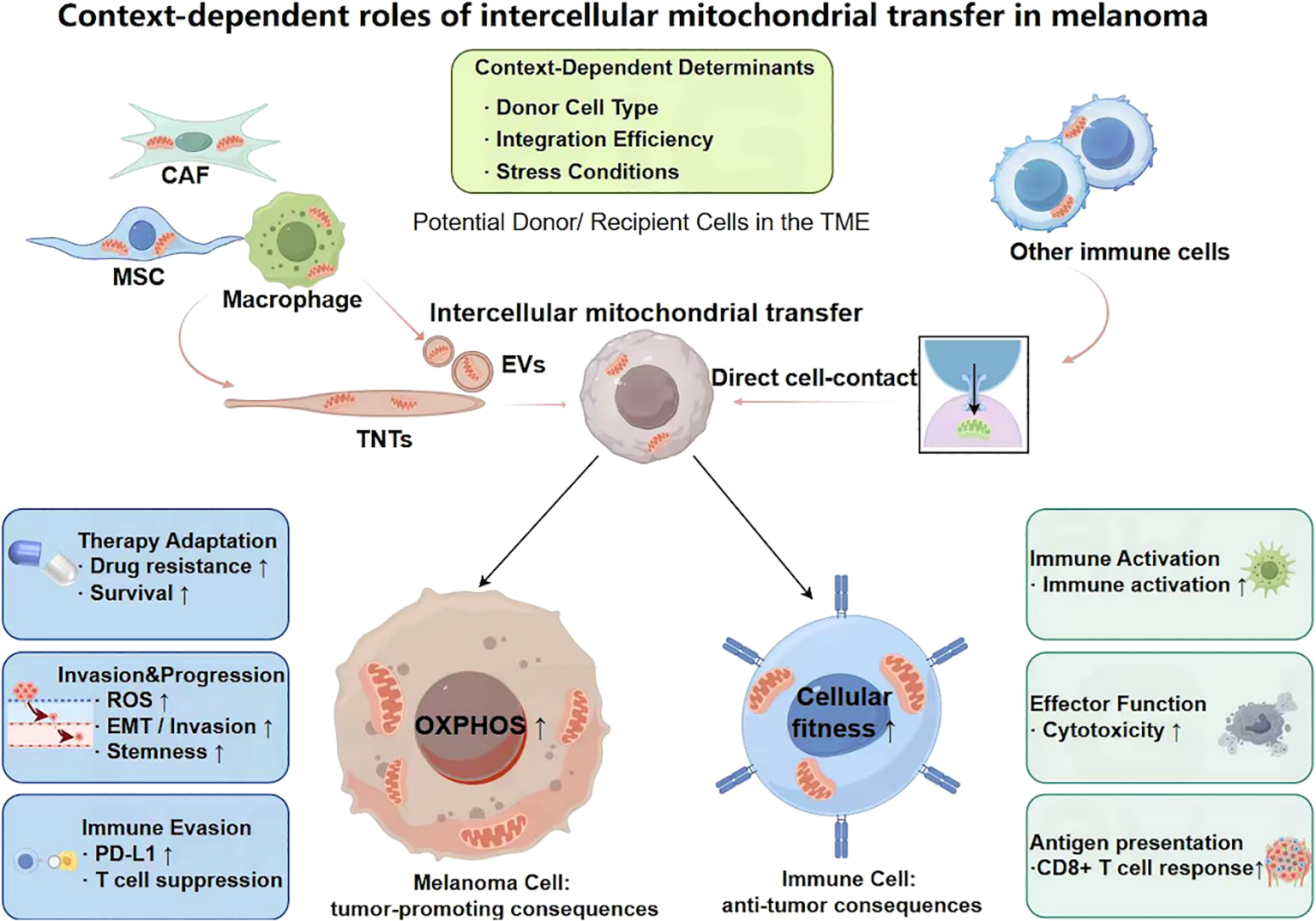
Dual functional roles of intercellular mitochondrial transfer in melanoma. Intercellular mitochondrial transfer exerts bidirectional effects on tumor and immune compartments within the melanoma microenvironment. In melanoma cells, acquired mitochondria enhance OXPHOS and metabolic fitness, thereby promoting tumor progression, including therapy resistance, immune evasion, and invasive capacity. In contrast, mitochondrial transfer to immune cells improves cellular fitness and supports antitumor responses, including enhanced antigen presentation and CD8^+^ T cell–mediated immunity. These functional outcomes are further modulated by donor cell type, integration efficiency, and cellular stress conditions.

In melanoma, intercellular mitochondrial transfer is increasingly recognized as a key regulatory mechanism shaping tumor behavior. As illustrated in **Figure 2**, this process exerts context-dependent effects across different cellular compartments within the tumor microenvironment. Under conditions of therapeutic pressure, hypoxia, or metabolic impairment, tumor cells can acquire functional mitochondria from surrounding stromal or neighboring cells via tunneling nanotubes or extracellular vesicles, thereby enhancing stress tolerance (70–72). Mitochondria and their associated components may also, under specific conditions, participate in the regulation of antitumor immune responses (73). Thus, intercellular mitochondrial transfer in melanoma does not represent a unidirectional tumor-promoting process; rather, it exhibits context-dependent, bidirectional functional effects across different cell types and microenvironmental conditions. These functional outcomes are jointly influenced by the identity of donor and recipient cells, the metabolic state of the recipient, and the therapeutic context.

### 4.1 Tumor-promoting effects in melanoma

#### 4.1.1 Energy restoration and therapeutic resistance

Melanoma exhibits pronounced metabolic plasticity, with tumor cells dynamically shifting between glycolysis and OXPHOS under therapeutic pressure (74). During treatment with BRAF/MEK inhibitors, melanoma cells transition toward a highly OXPHOS-dependent state, enhancing ATP production, maintaining mitochondrial membrane potential, and increasing anti-apoptotic capacity. This metabolic reprogramming reduces reliance on MAPK signaling and facilitates the development of acquired resistance (74–76). In the early phases of treatment or during acute stress, mitochondrial function in tumor cells may be transiently compromised (77). However, intercellular mitochondrial transfer can provide functional compensation for metabolically impaired cells.

Within the melanoma microenvironment, cancer-associated fibroblasts (CAFs), mesenchymal stem cells (MSCs), and specific immune cell populations may serve as potential mitochondrial donors (78, 79). Among immune cells, macrophages are the most plausible candidates, given their abundance within the tumor microenvironment, close physical interaction with tumor cells, and pronounced metabolic plasticity (80, 81). In addition, neutrophils may also participate in mitochondria-associated intercellular exchange under inflammatory conditions, although direct evidence in melanoma remains limited (82). Dendritic cells and T cells have likewise been implicated in mitochondria-dependent immune communication in other pathological settings, suggesting that they may contribute to donor-like functions under specific microenvironmental stresses (83, 84). However, the relative contribution, transfer route, and functional dominance of these immune cell subsets as mitochondrial donors to melanoma cells remain incompletely defined. Mouse studies have demonstrated that acquisition of exogenous mitochondria restores respiratory chain function, enhances ATP production, and re-establishes the NAD^+^/NADH ratio and mitochondrial membrane potential in recipient cells, thereby improving tolerance to therapeutic stress (85, 86). Restoration of mitochondrial function may further sustain ROS-dependent signaling, supporting continued activation of PGC1α-driven mitochondrial biogenesis and OXPHOS-related transcriptional programs (87–89).

#### 4.1.2 Immune evasion

Although melanoma exhibits responsiveness to immune checkpoint inhibitors, metabolic competition and alterations in the redox microenvironment are increasingly recognized as critical determinants of immunotherapy efficacy. Enhanced mitochondrial function, through the supplementation of functional respiratory chain components and restoration of electron transport capacity, provides metabolic support to compromised melanoma cells (90, 91). Following the acquisition of exogenous mitochondria, tumor cells gain a competitive advantage under hypoxic and nutrient-limited conditions, increasing oxygen consumption and reshaping the local redox environment. This, in turn, constrains the metabolic adaptability of T cells, impairing their proliferation and cytotoxic activity (92, 93).

Concurrently, mitochondrial-derived changes in ROS may regulate the expression of immunosuppressive molecules such as PD-L1 via redox-sensitive transcription factors and hypoxia-associated signaling pathways, further dampening antitumor immunity (94, 95). In addition, mtDNA, acting as a damage-associated molecular pattern, can modulate inflammatory signaling through pathways such as cGAS–STING, thereby influencing the magnitude and persistence of inflammation within the tumor microenvironment (96). In chronic tumor settings, this sustained, low-grade inflammatory activation may promote immune cell exhaustion or drive the expansion of immunosuppressive cell populations (97). Although direct evidence for immune cell participation in intercellular mitochondrial transfer in melanoma remains limited, emerging studies suggest that mitochondria-associated signal exchange may contribute to the establishment of an immunosuppressive metabolic niche (98).

#### 4.1.3 Enhancement of invasive capacity

Intercellular mitochondrial transfer may further promote melanoma cell invasion and metastasis through coordinated metabolic–signaling coupling. Following the acquisition of exogenous mitochondria, recipient cells exhibit enhanced OXPHOS activity and ATP production, accompanied by alterations in the redox environment (99). This suggests a close link between mitochondrial functional status and invasive phenotypes. Subsequent studies have shown that increased ROS levels can modulate the activity of EMT-associated transcription factors, while promoting matrix metalloproteinase expression and cytoskeletal remodeling, thereby enhancing migratory and invasive capacities (100, 101).

In this context, mitochondrial dynamics and spatial redistribution constitute key intermediates linking metabolic state to invasive behavior. Under conditions of increased mitochondrial fission, mitochondria preferentially localize to the leading edge of the cell, supplying localized ATP to support lamellipodia formation and focal adhesion turnover (102, 103). Disruption of DRP1-mediated mitochondrial fission significantly impairs tumor cell motility (104), further supporting a functional link between intercellular mitochondrial transfer and invasive behavior. In mouse models, enhanced mitochondrial function is frequently associated with increased metastatic burden, reinforcing its role in promoting tumor progression (105, 106).

### 4.2 Tumor-suppressive effects in melanoma

#### 4.2.1 Enhancement of antitumor immunity

In contrast to tumor cells, when immune cells serve as recipients of mitochondrial transfer, the functional consequences may diverge markedly. The acquisition of functional mitochondria can restore or enhance OXPHOS capacity in immune cells, supporting ATP production and redox homeostasis, thereby providing the metabolic foundation required for immune activation and sustained responses (107–109). Within the adaptive immune compartment, T cell function is highly dependent on dynamic metabolic programming. Persistent antigen stimulation or suppression within the tumor microenvironment can impair mitochondrial function, manifested by reduced membrane potential and redox imbalance, ultimately limiting T cell proliferation and cytotoxicity (110). Through mitochondrial transfer, restored OXPHOS capacity can enhance metabolic flexibility and promote differentiation toward memory-like or stem-like T cell states, thereby supporting durable antitumor immunity (111).

Beyond adaptive immunity, mitochondrial metabolism also exerts broad regulatory effects on innate immune cells. Animal studies have demonstrated that changes in OXPHOS activity and ROS levels influence macrophage polarization, supporting either pro-inflammatory or immunosuppressive phenotypes, and thereby generating context-dependent immunoregulatory effects within the tumor microenvironment (112, 113).

#### 4.2.2 Activation of innate immunity and type I interferon responses

Intercellular mitochondrial transfer may also activate innate immune sensing pathways through the exposure or aberrant localization of mitochondrial-derived components, thereby enhancing antitumor inflammatory responses. Following uptake of exogenous mitochondria, structural instability or incomplete integration may result in the release of mtDNA into the cytosol (114). Due to its bacterial-like features and unmethylated CpG motifs, mtDNA can be recognized by pattern recognition receptors and function as a potent damage-associated molecular pattern (115).

At the level of innate immunity, cytosolic mtDNA can activate the STING signaling axis, inducing the production of type I interferons and related inflammatory mediators (116, 117). Type I interferons not only promote dendritic cell maturation and antigen cross-presentation but also enhance the expansion, infiltration, and effector function of CD8^+^ T cells (118, 119). In melanoma, activation of the STING pathway is closely associated with intratumoral CD8^+^ T cell infiltration and improved responses to immune checkpoint blockade (120). In addition, mtDNA can engage TLR9 or the NLRP3 inflammasome to regulate inflammatory responses, inducing the release of cytokines such as IL-1β and promoting immune cell recruitment and remodeling of the tumor microenvironment (115, 121, 122). Under conditions of mitochondrial stress or damage, mtDNA and other mitochondrial-derived molecules may act as danger signals, enhancing dendritic cell cross-presentation and antigen processing efficiency, thereby strengthening tumor-specific T cell responses (123).

#### 4.2.3 Induction of tumor cell apoptosis

Mitochondria are not only central hubs of cellular energy metabolism but also key regulators of the intrinsic apoptotic pathway. Mitochondrial outer membrane permeabilization (MOMP) represents a critical point of no return in irreversible cell death, governed by BAX/BAK-mediated pore formation and the dynamic balance between pro- and anti-apoptotic proteins (124, 125). During intercellular mitochondrial transfer, exogenous mitochondria entering recipient cells do not always integrate stably into the endogenous mitochondrial network. When donor mitochondria are in a stressed state, or when metabolic activity and membrane potential are mismatched between donor and recipient mitochondria, electron transport efficiency may decline and mitochondrial heterogeneity may increase, leading to excessive ROS production (126). Elevated oxidative stress can activate BAX/BAK, promote MOMP, trigger cytochrome c release and downstream caspase cascades, and ultimately execute apoptotic cell death (127, 128).

In melanoma, the BRAF-driven MAPK signaling pathway is closely linked to mitochondrial homeostasis. BRAF/MEK inhibitors can induce mitochondrial stress during the early phases of treatment, rendering tumor cells relatively vulnerable (129). In this context, if intercellular mitochondrial transfer fails to achieve effective metabolic integration—or further increases mitochondrial burden—it may lower the apoptotic threshold and potentiate drug-induced cell death (126). In addition, mitochondria-mediated apoptosis may exhibit immunogenic features. Upon MOMP, mitochondrial-derived molecules such as mtDNA and ATP can be released into the extracellular space and recognized by the immune system, thereby promoting dendritic cell recruitment and antigen cross-presentation (130–132). This process can further activate tumor-specific T cell responses, characteristic of immunogenic cell death.

### 4.3 Key determinants governing the pro- or antitumor effects of intercellular mitochondrial transfer

Intercellular mitochondrial transfer does not confer a fixed biological outcome; rather, its functional consequences are largely determined by the type of recipient cell and are jointly modulated by mitochondrial integration status and the degree of microenvironmental stress. The same transfer event may elicit distinct metabolic and signaling responses depending on the cellular context. At the recipient level, when mitochondria are transferred into metabolically impaired melanoma cells, they typically restore oxidative phosphorylation and ATP production, thereby enhancing resistance to therapeutic stress (133). In contrast, when immune cells receive mitochondrial support, the predominant outcome is improved metabolic stability and enhanced effector function (68, 69).

At the level of mitochondrial integration, the efficiency of fusion between exogenous and endogenous mitochondrial networks further dictates functional outcomes. Efficient integration supports redox balance and energy metabolism, whereas impaired integration or stressed donor mitochondria may lead to ROS accumulation and membrane potential disruption, thereby altering cellular survival thresholds (126, 129). Meanwhile, microenvironmental stress and therapeutic conditions shape the direction of downstream signaling responses. Under mild stress, mitochondrial supplementation generally provides metabolic support (134). However, under sustained hypoxia, nutrient deprivation, or targeted therapy, additional mitochondrial burden may amplify oxidative stress or innate immune signaling, shifting the balance toward apoptosis or immune activation (135, 136).

Importantly, both the occurrence and directionality of intercellular mitochondrial transfer exhibit pronounced context dependency. Distinct microenvironmental stresses and cellular states may determine not only the transfer route but also its functional consequences. Under hypoxic or acute injury conditions, cells tend to favor rapid and directional mitochondrial transfer via TNTs to achieve local energy compensation (20, 137). In contrast, under inflammatory or chronic stress conditions, EV-mediated transfer of mitochondrial components may become more prominent, facilitating long-distance signaling (138). Additionally, differences in metabolic status between donor and recipient cells can drive transfer directionality: metabolically intact stromal cells often supply mitochondria to metabolically compromised tumor cells (139), whereas under immune-activating conditions, immune cells themselves may act as recipients, enhancing their functional capacity (140).

Furthermore, intercellular mitochondrial transfer may regulate its functional outcomes through mitochondrial quality control mechanisms. Following entry into recipient cells, a subset of exogenous mitochondria—particularly those that are functionally impaired or poorly integrated—may be recognized and targeted for selective clearance via PINK1/Parkin-mediated mitophagy (141, 142). This process partially determines the effective contribution of transferred mitochondria to cellular function. Moderate levels of mitophagy help maintain mitochondrial integrity and support metabolic stability under stress conditions (143). However, when mitochondrial influx or damage exceeds the capacity of quality control systems, accumulation of dysfunctional mitochondria and elevated ROS levels may occur, ultimately altering cell fate (144). Thus, mitophagy may serve as a critical regulatory node influencing the functional direction of intercellular mitochondrial transfer in melanoma, with implications for metabolic adaptation and therapeutic response.

## 5 Therapeutic potential and challenges of intercellular mitochondrial transfer in melanoma

The significance of intercellular mitochondrial transfer in melanoma lies primarily in its role in shaping tumor adaptive evolution. Under therapeutic pressure, this process can provide metabolic compensation to tumor cells and stabilize resistant subpopulations; conversely, under conditions of impaired mitochondrial integration or heightened stress, it may lower the apoptotic threshold and enhance immune recognition. This functional plasticity positions intercellular mitochondrial transfer as a critical node linking metabolic adaptation, immune regulation, and therapeutic response. To systematically delineate its context-dependent effects on disease outcomes, we have integrated its functional roles across different conditions (see **Table 2**).

**Table 2:** Context-dependent functional roles of intercellular mitochondrial transfer in melanoma.

Context	Key molecules/mechanisms	Impact on melanoma outcomes	References
Early phase of BRAF/MEK inhibitor treatment	MITF–PGC1α axis, Miro1, DRP1	Promotes the development of acquired resistance	(35, 145, 146)
Acute metabolic stress or mitochondrial dysfunction	Respiratory chain complex integration	Enhances cell survival and delays therapeutic response	(147, 148)
Formation of high-OXPHOS subpopulations	PGC1α, mitochondrial biogenesis pathways	Stabilizes resistant subpopulations and promotes disease progression	(149, 150)
Hypoxic or chronically inflamed microenvironment	ROS signaling, EMT-related pathways	Enhances invasive and metastatic potential	(151, 152)
Impaired mitochondrial integration or transfer of damaged mitochondria	BAX/BAK, MOMP pathway	Lowers apoptotic threshold and increases drug sensitivity	(153, 154)
Mitochondrial stress with mtDNA release	cGAS–STING axis	Enhances immune recognition and therapeutic responsiveness	(155, 156)

### 5.1 Therapeutic potential

In melanoma development and treatment, intercellular mitochondrial transfer exhibits pronounced context dependency. Its therapeutic value does not lie in simply inhibiting or enhancing the process per se, but rather in selectively targeting distinct regulatory layers. Emerging evidence indicates that under therapeutic pressure—such as BRAF/MEK inhibition—metabolically compromised melanoma cells can restore OXPHOS, sustain ATP production, and maintain redox balance by acquiring exogenous mitochondria. This facilitates the selective survival of high-OXPHOS subpopulations and contributes to the development of acquired resistance (35, 145, 146). In multiple tumor models, enhanced mitochondrial function has also been closely associated with ROS-mediated signaling, transcriptional reprogramming, and the maintenance of immunosuppressive phenotypes (154–156).

Accordingly, current therapeutic strategies targeting intercellular mitochondrial transfer primarily focus on two interrelated levels. The first involves direct interference with the transfer process itself. Preclinical studies have shown that inhibiting cytoskeletal remodeling or disrupting TNT formation can reduce mitochondrial trafficking between cells, thereby limiting the ability of tumor cells to acquire exogenous metabolic support (22, 25). In addition, modulation of mitochondrial transport-related proteins or interference with EV release and uptake may also affect the intercellular transfer of mitochondrial components (21, 157). However, these approaches remain largely confined to *in vitro* and animal studies, and direct translational evidence in melanoma is still limited.

The second strategy targets the metabolic dependencies established following mitochondrial acquisition. Evidence suggests that tumors receiving exogenous mitochondria often exhibit increased reliance on OXPHOS, and inhibition of the mitochondrial respiratory chain can reduce tumor cell viability and delay the emergence of resistance-associated phenotypes in preclinical models (154, 155). Compared with directly blocking mitochondrial transfer, exploiting these induced metabolic vulnerabilities may represent a more feasible therapeutic approach. Nevertheless, such strategies are currently framed within broader interventions targeting tumor metabolic reprogramming and cannot yet be considered specific therapies directed at intercellular mitochondrial transfer.

Importantly, under certain stress conditions, intercellular mitochondrial transfer may also exert tumor-suppressive effects. When exogenous mitochondria are functionally impaired or poorly integrated, the resulting mitochondrial burden can lead to ROS accumulation and disruption of membrane potential, thereby inducing mitochondrial outer membrane permeabilization and triggering apoptosis (153). In the context of targeted therapy or chemotherapy, this additional metabolic stress may amplify drug-induced cell death signals, converting a compensatory mechanism into an apoptotic amplification pathway (156).

Moreover, mitochondrial stress-associated release of mtDNA can activate the cGAS–STING signaling axis, inducing type I interferon production and enhancing antigen presentation, thereby improving responses to immune checkpoint inhibitors (96, 156). In parallel, enhancing mitochondrial function in immune cells may also hold therapeutic promise. For instance, maintaining OXPHOS capacity and metabolic stability in T cells can support their effector function and memory differentiation (110, 123). However, these strategies remain at an exploratory stage, and their specificity and safety profiles have yet to be fully defined.

### 5.2 Clinical challenges

Despite its therapeutic potential, targeting intercellular mitochondrial transfer in melanoma faces substantial challenges at the clinical level. At present, direct evidence for the occurrence and functional relevance of this process in patients remains limited, with a lack of systematic histopathological validation and prospective clinical studies (21, 157). From an implementation perspective, a primary challenge is the lack of intervention specificity. Mitochondrial transfer may exert opposing effects in tumor cells and immune cells; thus, how to inhibit tumor-supportive metabolic compensation without compromising immune cell function remains an unresolved and critical issue.

In addition, there are currently no clinically applicable biomarkers capable of directly monitoring intercellular mitochondrial transfer, which constrains both patient stratification and the evaluation of therapeutic efficacy. The optimal timing of intervention also remains uncertain, as the functional role of mitochondrial transfer may vary across different stages of treatment. Furthermore, melanoma exhibits pronounced heterogeneity in metabolic dependency, OXPHOS activity, and immune infiltration, leading to considerable variability in patient responses to mitochondria-targeted interventions (117, 146, 154). Finally, from a safety perspective, mitochondria are fundamental to energy metabolism across normal tissues; therefore, interventions targeting mitochondrial dynamics or OXPHOS function may carry the risk of systemic metabolic toxicity (158).

## 6 Conclusion and future perspective

Intercellular mitochondrial transfer is more likely to represent an adaptive regulatory mechanism employed by melanoma under therapeutic pressure. By reshaping the functional interplay between metabolism and immunity, this process plays a critical role in tumor survival, the development of therapeutic resistance, and the modulation of the tumor microenvironment. Current evidence indicates that mitochondrial transfer can support tumor cell metabolism by enhancing OXPHOS, while under specific stress conditions it may also influence cell fate through the induction of mitochondrial damage signals and activation of immune-related pathways. Importantly, its functional consequences are not fixed but are determined by multiple factors, including the type of recipient cell, the efficiency of mitochondrial integration, and the surrounding microenvironmental context.

Future research should move beyond the binary classification of intercellular mitochondrial transfer as either tumor-promoting or tumor-suppressive, and instead focus on elucidating the key regulatory determinants that govern its functional direction. In particular, differential responses across cell types and dynamic changes across treatment stages are likely to be critical factors influencing therapeutic outcomes. Building on mechanistic insights, there is a need to establish biomarker systems capable of capturing the activity and functional state of mitochondrial transfer. Such advances would enable the development of stratified and temporally optimized intervention strategies. Integrating approaches targeting MAPK signaling, OXPHOS regulation, and immunotherapy may ultimately improve the overall efficacy of melanoma treatment.
